# Correlates of objectively measured sedentary time and self-reported screen time in Canadian children

**DOI:** 10.1186/s12966-015-0197-1

**Published:** 2015-03-18

**Authors:** Allana G LeBlanc, Stephanie T Broyles, Jean-Philippe Chaput, Geneviève Leduc, Charles Boyer, Michael M Borghese, Mark S Tremblay

**Affiliations:** Healthy Active Living and Obesity Research Group, Children’s Hospital of Eastern Ontario Research Institute, 401 Smyth Road, Ottawa, Ontario K1H 8 L1 Canada; Population Health, Faculty of Graduate and Postdoctoral Studies, University of Ottawa, Ottawa, Ontario Canada; Pennington Biomedical Research Center, Baton Rouge, Louisiana US; Department of Pediatrics, Faculty of Medicine, University of Ottawa, Ottawa, Ontario Canada; School of Human Kinetics, Faculty of Health Sciences, University of Ottawa, Ottawa, Ontario Canada

**Keywords:** Sedentary behaviour, Television viewing, Pediatric

## Abstract

**Background:**

Demographic, family, and home characteristics play an important role in determining childhood sedentary behaviour. The objective of this paper was to identify correlates of total sedentary time (SED) and correlates of self-reported screen time (ST) in Canadian children.

**Methods:**

Child- and parent-reported household, socio-demographic, behavioural, and diet related data were collected; directly measured anthropometric and accelerometer data were also collected for each child. Participants with complete demographic, anthropometric, and either SED (n=524, 41% boys) or ST (n=567, 42% boys) data from the Canadian site of the International Study of Childhood Obesity Lifestyle and the Environment (ISCOLE) were included in analysis. Sixteen potential correlates of SED and ST were examined using multilevel general linear models, adjusting for sex, ethnicity, number of siblings, and socio-economic status. All explanatory variables moderately associated (p<0.10) with SED and/or ST in univariate analyses were included in the final, fully-adjusted models. Variables that remained significant in the final models (p<0.05) were considered correlates of SED and/or ST.

**Results:**

Children averaged 8.5hours of daily SED; no differences in total SED, or total ST were seen between girls and boys, but boys reported significantly more video game/computer usage than girls. Boys also had higher waist circumference and BMI z-scores than girls. In the final models, waist circumference and number of TVs in the home were the only common correlates of both SED and ST. SED was also negatively associated with sleep duration. ST was also positively associated with mother’s weight status, father’s education, and unhealthy eating pattern score and negatively associated with healthy eating pattern score, and weekend breakfast consumption. Few common correlates existed between boys and girls.

**Conclusion:**

Several factors were identified as correlates of SED and/or of ST in Canadian children; however, few correlates were common for both SED and ST, and for both boys and girls. This suggests that a single strategy to reduce SED and ST is unlikely to be effective. Future work should examine a variety of other, non-screen based sedentary behaviours and their potential correlates in the hopes of creating tailored public health messages to reduce SED and ST in both boys, and girls.

## Introduction

Sedentary time (SED) is characterized by waking behaviours that require little energy expenditure (i.e. ≤1.5 METs) and that occur in a sitting or reclined position [1]. SED can be further classified by a variety of sedentary behaviours such as reading, playing quietly, watching television (TV), or using the computer. SED should be thought of as a separate and distinct behaviour from physical activity and not simply the failure to meet prescribed physical activity guidelines [2]. To help guide parents and caregivers, the Canadian Society for Exercise Physiology developed Canadian Sedentary Behaviour Guidelines for Children and Youth [3]. They provide a general recommendation for children and youth to minimize the time they spend sedentary each day; furthermore, they provide specific recommendations to limit recreational screen time to no more than 2 hours per day, and to limit sedentary (i.e., motorized) transport, extended time spent sitting, and time spent indoors throughout the day [3].

Previously, research examining SED has been largely informed by parent- and child-report questionnaires, which focus primarily on specific sedentary behaviours, such as screen time (ST), rather than total daily SED. Results from a recent systematic review focusing on ST suggests that lower ST is associated with better measures of body composition, fitness, self-esteem, self-worth, pro-social behaviour, and academic achievement [4]. Now, with widespread use of accelerometers, researchers can examine total daily SED, including its effect on acute and chronic health conditions. In adults, higher total SED is associated with higher risk of cardiovascular disease, overweight/obesity, and premature mortality [5-7]. However, in children, the relationship between accelerometer measured total SED and ill-health is less evident, and possibly more complex. For example, evidence from both clinical and population based studies has shown that in children, long bouts of SED are not associated with acute elevations in cardio-metabolic health risk [8], body mass index (BMI), or waist circumference [9]. Further, previous work has shown that ST accounts for only a small proportion of SED and the appropriateness of using ST as a proxy measure for SED has been questioned [10]. Still, many studies use measures of SED and ST interchangeably, and make public health messages based on these mixed results.

A recent review by Temmel and Rhodes identified 64 studies examining correlates of sedentary behaviour however, only eleven reported on both accelerometer measured SED and self-reported ST in the same population, and none of the 64 studies included data from Canadian children [11]. Of the eleven studies examining both SED and ST, age, sex, ethnicity, socio-economic status, and day of the week (weekend versus weekday) were significant correlates of both SED and ST. Physical maturity was a correlate of SED only; whereas urban versus rural living, neighbourhood satisfaction and safety, access (e.g., to a television), sleep duration, and self-esteem were correlates of ST only. Other studies that have explored associations with ST have identified age, socio-economic status, single-parent households, ethnicity, and sex as important correlates [12,13]. Studies that have examined correlates of SED have shown significant associations with parental BMI, TV viewing, and computer use [14].

Identifying common correlates of SED and ST can help to inform public health strategies and messages to improve habitual behaviour in young people. The purpose of this study was to examine anthropometric, behavioural, parental, and household correlates of objectively measured SED and self-report ST in a sample of Canadian children. Analyses were grounded in the socio-ecological model for sedentary behaviour, as proposed by Owens et al. in order to understand multiple influences on behavior [15].

## Methods

### Data source

The International Study of Childhood Obesity, Lifestyle and the Environment (ISCOLE) aimed to determine the relationships between lifestyle behaviours and obesity in a multi-national, cross-sectional study of 10-year-old children [16]. ISCOLE was designed to allow researchers to investigate the influence of higher-order characteristics such as behavioural settings, and the physical, social and policy environments, within and between countries [16]. Additional details on study design, participating countries, and full methodology has been published elsewhere [16]. As Canada was the first country to develop evidence-informed sedentary behaviour guidelines [3], the analyses for the present study focused on data from ISCOLE Canada. Data collection occurred in Ottawa, Canada between September 2012, and May 2013 and included 26 schools, from four school boards: English Public, French Public, English Catholic, and French Catholic. All schools within each stratum were invited to participate and the first ones to respond were included into the study with a recruitment goal of 500 participants, in agreement with the rules of our research ethics boards; the response rate was 50% (children with consent to participate divided by envelopes distributed). This project was approved by the research ethics board at the Children’s Hospital of Eastern Ontario and the participating school boards. Written informed parental consent and child assent were obtained for all participants.

### Dependent variables

#### Accelerometer measured sedentary time

The ActiGraph GT3X+ triaxial accelerometer (ActiGraph LLC, Pensacola, FL, USA) was used to objectively measure total SED. Participants wore the accelerometer, attached to an elastic belt around the waist (at the right mid-axillary line), for 7 consecutive days, 24 hours per day, removing only for aquatic activities (e.g., bathing, swimming). To increase compliance, study staff instructed children how to wear the accelerometer during the initial in-school assessment, and conducted an in-person compliance check 2–4 days after initialization. Further, participants were contacted twice via telephone (one weekday evening and one weekday) to ensure they were wearing the device, and to address any questions.

To be included in the ISCOLE dataset, participants were required to provide at least four days of valid measurements (including at least one weekend day), with at least 10 hours of waking wear time per day [17,18]. Data were collected at a sampling rate of 80 Hz, downloaded in 1-second epochs, and aggregated to 15-second epochs [18]. Total sedentary time (SED) was defined as all epochs showing ≤25 counts per 15 seconds, consistent with widely used cutoffs from Evenson et al. [19] (i.e., sedentary: 0–25 counts/15 seconds, light: 26–573 counts/15 seconds, moderate-to-vigorous: ≥574 counts/15 seconds). For analysis, total SED was treated as a continuous variable.

#### Self-reported screen time

Child-reported screen time was determined from a Diet and Lifestyle Questionnaire, with questions taken from the U.S. Youth Risk Behaviour Surveillance System [16,20]. Children were asked how many hours they typically watched TV, and how many hours they played video games and/or used the computer per week day, and per weekend day [16]. Responses were: 1 = I did not watch TV, 2 = Less than 1 hour, 3 = 1 hour, 4 = 2 hours, 5 = 3 hours, 6 = 4 hours, 7 = 5 or more hours. Response categories were collapsed to include 1 = ≤1 hour of TV, 2 = 2 hours, 3 = 3 hours, 4 = 4 hours, and 5 = ≥5 hours. A weighted mean score of daily screen time was then calculated as follows: [(hours of TV on weekdays x 5) + (hours of TV on weekend days × 2) + (hours of video games and computers on weekdays × 5) + (hours of video games and computers on weekend days × 2)]/7. For analysis, this is presented as a screen time score, rather than total hours of ST since after 5 hours per day, we could not ascertain the participant’s actual amount of ST. Self-report methods of quantifying ST have been reported to have acceptable reliability and validity in children [21]. After testing for normality, ST score was log-transformed for analysis and treated as a continuous variable.

### Potential correlates

#### Anthropometric and biological variables

Anthropometric data (i.e., height, weight, waist circumference, percent body fat) were directly measured by trained ISCOLE data collectors during the in-school visit according to standardized procedures [16]. Height was measured to the nearest 0.1 cm using a Seca 213 portable stadiometer (Hamburg, Germany). Weight (to the nearest 0.1 kg) and body fat percentage (to the nearest 0.1%) were measured using a portable Tanita SC-240 Body Composition Analyzer (Arlington Heights, IL, USA). Participants were asked to remove heavy clothing, and objects from their pockets before stepping on the scale. The Tanita SC-240 has shown acceptable accuracy for estimating body fat percentage when compared with dual-energy X-ray absorptiometry, supporting its use in field-based studies [22]. Body mass index (BMI) was calculated (kg/m^2^), and BMI z-score, based on age and gender, was determined using the Centers for Disease Control and Prevention (CDC) growth charts [23] for all participants. Waist circumference was measured using a non-elastic anthropometric tape after a normal exhalation to the nearest 0.1 cm, at the mid-point between the top of the iliac crest and the bottom of the last floating rib. Children and parents were also asked about the child’s age, sex, birth country and ethnicity in the ISCOLE Diet and Lifestyle Questionnaire and the Demographic and Family History Questionnaire.

#### Behavioural characteristics

All children completed the ISCOLE Diet and Lifestyle Questionnaire, which included a compilation of validated questions from several questionnaires [16]. Children completed a Food Frequency Questionnaire (FFQ) adapted from the Health Behaviours in School-age Children (HBSC) study [24], which asked how often participants consumed 23 food items in a usual week. To identify existing dietary patterns among the study population, principal components analyses were carried out using the FFQ food groups as input variables (excluding fruit juices due to low validity) (unpublished analysis). Eigenvalues and a scree plot analysis were used as the criteria for deciding the number of factors extracted. The two criteria lead to similar conclusions and two factors were eventually chosen for each analysis. The factors were then rotated with an orthogonal varimax transformation to force non-correlation of the factors and to enhance the interpretation. Two factors were included in this manuscript as exposure variables: “unhealthy eating pattern score” (e.g., hamburgers, soft drinks, fried food; higher score means worse eating pattern) and “healthy eating pattern score” (e.g., vegetables and fruits; higher score means better eating pattern).

Children were asked how they got to school most days (e.g., walking, car), and these responses were re-coded as active or inactive transport. Child-reported breakfast consumption (weekday and weekend frequency), and sleep (quality and quantity) were also assessed as potential correlates. Physical activity was captured via self-report questionnaire to determine how many days per week children were active for at least 60 minutes.

#### Parent and home environment

The Demographic and Family History Questionnaire and the ISCOLE neighbourhood and home environment questionnaires were sent home with the child at the same time as the parent-consent forms for the parents to fill out (i.e. prior to child assessment) [16]. Demographic questionnaires completed by the parents captured additional information about the participating number of siblings, household income (8 options ranging from < $14,999, to > $140,000 or more), parental education (ranging from “less than high school” to “post graduate degree”), parental-report weight and height (used to calculate parental weight status), number of TVs in the home, and presence of a TV in the child’s bedroom. See Table 1 for additional details on response categories and additional details on measurement and analysis of all potential correlates.

**Table 1:** **Potential correlates of objectively measured sedentary time and self-reported sedentary behaviour**

**Variable**	**Measurement method**	**Use in analysis**
***Anthropometric***		
Sex	Parent-report: Demographic and Family History Questionnaire	Binary variable: male, female (used as a covariate)
BMI	Directly measured height and weight, calculated using CDC cut-points [23]	Ordered categorical: underweight, normal weight, overweight, obese
Percent body fat	Directly measured using Tanita	Continuous
Waist circumference	Directly measured by ISCOLE researcher	Continuous
Child ethnicity	Parent-report: Demographic and Family History Questionnaire	Categorical: white, African American, Asian, First Nations, East Indian, don’t know, other (used as a covariate)
***Behavioural characteristics***		
Healthy and unhealthy eating scores	Child-report food frequency questionnaire: ISCOLE Diet and Lifestyle Questionnaire	Continuous: Continuous: obtained from a principal component analysis derived from a 23-item food frequency questionnaire
Breakfast consumption (weekend, and weekday)	Child-report: ISCOLE Diet and Lifestyle Questionnaire	Re-coded as dichotomous: those who ate breakfast at least once per weekday (versus never) and those who at breakfast at least weekend day (versus never).
Commute to school (main part of journey)	Child-report: ISCOLE Diet and Lifestyle Questionnaire	Re-coded as dichotomous: active (walking, bicycle/rollerblade/skateboard/scooter, other), and inactive (bus/train/tram/underground/boat, car/motorcycle/moped)
Sleep (in the past week)	Child-report: ISCOLE Diet and Lifestyle Questionnaire	Categorical: Sleep quality (4 responses: very good, fairly good, fairly bad, very bad), sleep quantity (4 responses: very good, fairly good, fairly bad, very bad). Re-coded for analysis, collapsing very good and fairly good categories, and fairly bad and very bad categories for both sleep quality and sleep quantity.
Physical activity	Child-report: ISCOLE Diet and Lifestyle Questionnaire	Categorical: 8 responses (0 days, 1 day, 2 days, 3 days, 4 days, 5 days, 6 days, 7 days). Was included in the model re-coded as those active more and less than 6 days in the past week.
	Accelerometer measured minutes per day of LPA and MVPA	Accelerometer: time spent at different PA intensities using Evensen cut-points [19]
***Family situation***		
Number of siblings	Parent-report: Demographic and Family History Questionnaire	Continuous (used as a covariate)
Parental BMI	Parent-report: Demographic and Family History Questionnaire	Re-coded as dichotomous: normal weight, or overweight/obese
Parental education	Parent-report: Demographic and Family History Questionnaire	Re-coded as dichotomous: ≤high school, and high school or higher
Combined household income	Parent-report: Demographic and Family History Questionnaire	Ordered categorical: 8 options ranging from < $14,999, to > $140,000 or more (used as covariate). In the model, income was re-coded as above/below mean category.
***Home environment***		
Number of TVs in home	Parent report: ISCOLE neighbourhood and home environment questionnaire	Re-coded as dichotomous: ≤1, and ≥2
TV/electronics in bedroom	Child-report: ISCOLE Diet and Lifestyle Questionnaire	Binary response: yes/no
	Parent report: ISCOLE neighbourhood and home environment questionnaire	
Automobile ownership	Parent report: ISCOLE neighbourhood and home environment questionnaire	Continous: number of working automobiles owned per household. Re-coded as dichotomous: <2 and ≥2.

BMI: body mass index; ISCOLE: International Study of Childhood Obesity Lifestyle and the Environment; LPA: light-intensity physical activity; MVPA: moderate- to-vigorous-intensity physical activity; PA: physical activity.

#### Statistical analysis

Statistical analyses were conducted using SAS 9.4 (SAS Institute Incorporated, North Carolina, USA). Descriptive information (mean, standard deviation) was calculated for demographic and anthropometric characteristics of all participants and their parents. Unpaired t-tests and chi-squared tests were run to determine differences between boys and girls.

Multilevel general linear models (PROC MIXED), including school as a random effect, were used to determine correlates of SED and ST. Multilevel models properly account for the hierarchical nature of the data. Potential correlates were first included in univariate models; variables that were significant, or marginally significant (p < 0.10), were subsequently included in domain-specific models similar to those outlined in the social ecological model proposed by Owen et al. for sedentary behaviour [15] (i.e., demographic/biological characteristics, family characteristics, home environment characteristics, or behavioural characteristics). Sex and ethnicity were included as covariates for all univariate models. Variables that were marginally significantly (p < 0.10) correlated with SED or ST in the domain-specific models were included in the final model. Variables that remained significant (p < 0.05) in the final model were considered correlates of SED and/or ST. Sex, ethnicity, number of siblings, and household income were used as covariates for all multivariable models. These covariates were selected based on the plausibility of their potential confounding effect and because of their known associations with sedentary behaviour. The Kenward-Roger approximation (DDFM = KR) was used to calculate degrees of freedom [25]. Analyses were conducted separately for the total sample, boys, and girls. Multicollinearity was tested using tolerance and variance inflation factors, and results indicated no issues with multicollinearity [26].

## Results

### Descriptive characteristics

In total, 567 (mean age = 10.0, 42.3% boys) participants provided complete data and were included in analysis. Participants were from the English Public (n = 393; 69.3% of total sample), French Public (n = 60; 10.6% of total sample), English Catholic (n = 75; 13.2% of total sample) and French Catholic (n = 39; 6.8% of total sample) School Boards. Participants were born in 35 different countries, with the majority (88.9%) being born in Canada, and declaring themselves as “white/Caucasian” (66.6%). The majority of children had either one (51.3%) or two (23.7%) siblings. Table 2 shows the frequency and distribution of potential correlates of sedentary behaviour between boys and girls.

**Table 2:** **Participant characteristics (mean (SD), unless otherwise noted)**

	**Total (** ***n*** **= 567)**	**Boys (** ***n*** **= 239)**	**Girls (** ***n*** **= 328)**
***Child characteristics***			
Age (years)	10.0 (0.4)	10.1 (0.4)	10.0 (0.4)
Height (cm)	143.8 (7.2)	143.6 (6.8)	143.9 (7.4)
Weight (kg)	38.1 (9.0)	38.3 (9.1)	37.9 (9.1)
Percent body fat	20.5 (7.4)	18.7 (7.2)	21.9 (7.3)*
Waist circumference (cm)	63.0 (8.4)	64.1 (8.8)	62.2 (8.0)**
BMI *z*-score	0.20 (1.02)	0.32 (0.98)	0.11 (1.04)***
Weight status (*n*,%)			
Underweight	15 (2.7%)	2 (0.8%)	13 (4.0%)
Normal weight	422 (74.7%)	177 (74.4%)	245 (74.9%)
Overweight	68 (12.0%)	29 (12.2%)	39 (11.9%)
Obese	60 (10.6%)	30 (12.6%)	30 (9.2%)
Ethnicity (*n*,%)			
White/Caucasian	373 (66.6%)	160 (68.1%)	213 (65.6%)
African American	15 (2.7%)	4 (1.7%)	11 (3.4%)
Asian	57 (10.2%)	25 (10.6%)	32 (9.9%)
First Nations	2 (0.4%)	1 (0.4%)	1 (0.3%)
East Indian	5 (0.9%)	1 (0.4%)	4 (1.2%)
Do not know/other	108 (19.3%)	11 (18.7%)	64 (19.6%)
Physical activity (min/day)			
MVPA	58.7 (19.3)	67.1 (19.3)	52.7 (17.0)*
Moderate	41.8 (12.1)	47.6 (11.7)	37.8 (10.7)*
Light PA	304.8 (45.0)	310.1 (43.7)	301.1 (45.5)****
Total SED (min/day)	511.4 (63.1)	506.9 (66.0)	514.5 (60.9)
Self-reported screen time score (hour/day)			
Total screen time	2.8 (1.8)	2.9 (1.6)	2.7 (1.9)
TV	1.5 (1.3)	1.5 (1.1)	1.6 (1.2)
Video game/computer	1.3 (1.0)	1.4 (1.0)*	1.1 (1.0)
Household income (*n*,%)			
Less than $14,000	16 (3.0)	5 (2.2)	11 (3.5)
$15,000--59,999	89 (16.4)	33 (14.4)	56 (17.7)
$60,000-139,999	231 (42.4)	100 (43.7)	131 (39.4)
$140,000 and above	209 (38.4)	91 (39.7)	118 (37.3)
***Parental characteristics***			
***Mother***			
Education			
High school or less	85 (15.3)	35 (15.1)	50 (15.4)
Greater than high school	473 (84.7)	198 (85.0)	275 (84.6)
Self-reported BMI (kg/m^2^)	24.9 (5.2)	25.3 (5.6)	24.6 (4.9)
Age (years)	41.7 (5.1)	41.5 (4.9)	41.9 (5.2)
***Father***			
Education			
High school or less	102 (18.7)	40 (17.7)	56 (19.3)
Greater than high school	444 (81.3)	185 (82.3)	259 (80.7)
Self-reported BMI (kg/m^2^)	26.8 (4.4)	26.7 (4.4)	26.8 (4.4)
Age (years)	44.1 (6.0)	44.4 (6.2)	43.8 (5.8)

BMI: body mass index; MVPA: moderate- to-vigorous-intensity physical activity; PA: physical activity; SED: accelerometer measured total daily sedentary time.

Unpaired t-test * p < 0.0001, ** p = 0.0069, ***p = 0.0175, ****p = 0.0245.

The majority of mothers were normal weight (64.8%) and had above a high school education (84.7%); the majority of fathers were overweight or obese (56.8%) and had above a high school education (81.3%). Most parents reported having two or more TVs in their house (96.2%) with the remainder reporting having either no TV, or one TV; the majority subscribed to cable + premium channels (39.6%) with cable internet service (59.1%). No differences were seen in parental characteristics between boys and girls.

Most children ate breakfast every weekday (82.9%) and every weekend day (89.8%). The majority of children reported that they had “very good” or “fairly good” sleep quality (91.4%) and sleep quantity (90.8%). On average, ISCOLE participants accumulated an average of 511.4 (63.1) minutes of total daily SED (approximately 8.5 hours) and had a ST score of 2.8 (1.8) (approximately 2.8 hours of average daily total TV, computer, and video game use). Total ST did not differ between boys and girls, but boys reported playing more video games than girls.

### Univariate analyses

The results of the univariate regression analyses are presented in Tables 3 and 4. Of the 16 potential behavioural, parental, and home correlates, 5 were significantly associated with SED in the total sample (4 for boys, and 7 for girls) and 11 were significantly associated with ST in the total sample (10 for boys, and 9 for girls).

**Table 3:** **Univariate correlates of total sedentary time** ^**a**^

	**Total**			**Boys**			**Girls**		
**Variables**	**β-coefficient**	**SE**	***p-*** **value**	**β-coefficient**	**SE**	***p-*** **value**	**β-coefficient**	**SE**	***p-*** **value**
***Anthropometric*** ^***b***^									
Percent body fat	**1.62**	**0.37**	**<0.0001**	**0.48**	**0.62**	**0.0172**	**1.57**	**0.48**	**0.0012**
Waist circumference	**1.66**	**0.31**	**<0.0001**	**1.50**	**0.47**	**0.0017**	**1.64**	**0.43**	**0.0001**
BMI z-score	**7.61**	**2.63**	**0.0039**	7.00	4.42	0.1147	**6.93**	**3.30**	**0.0366**
***Behavioural characteristics***									
Unhealthy eating score	**−6.08**	**2.87**	**0.0347**	−5.33	3.70	0.1507	**−8.38**	**4.12**	**0.0830**
Healthy eating score	−2.47	2.71	0.3627	−3.33	4.43	0.4527	0.25	3.51	0.9429
Weekday breakfast consumption	**30.42**	**18.15**	**0.0949**	−10.07	43.28	0.8162	**35.95**	**20.00**	**0.0727**
Weekend breakfast consumption	33.34	20.36	0.1022	27.49	33.10	0.4073	40.10	26.72	0.1346
Commute to school	0.25	6.43	0.9688	−1.87	9.75	0.8482	−0.21	8.55	0.9850
Sleep quality	9.42	10.00	0.3467	8.37	17.54	0.6339	13.15	12.36	0.2883
Sleep quantity	**26.04**	**9.31**	**0.0053**	**30.12**	**14.28**	**0.0361**	**27.99**	**12.81**	**0.0296**
Self-reported PA	8.00	5.69	0.1620	1.27	9.08	0.8889	**12.72**	**7.50**	**0.0907**
***Family situation***									
Mother weight status	−2.99	6.02	0.6197	−2.47	9.52	0.7955	−3.33	7.86	0.6726
Father weight status	−1.43	5.57	0.7968	−5.29	8.88	0.5524	−0.23	7.18	0.9747
Mother education	−1.06	8.07	0.8961	−4.18	13.22	0.7525	2.53	10.10	0.8028
Father education	−4.66	7.24	0.5202	−10.83	11.65	0.3535	1.14	9.28	0.9025
Household income	−5.33	5.75	0.3569	−12.46	8.98	0.1670	−2.14	7.55	0.7773
***Home environment***									
# of TV’s in home	**16.24**	**6.17**	**0.0087**	**20.26**	**9.91**	**0.0422**	**13.89**	**7.95**	**0.0816**
TV in bedroom	−8.05	7.62	0.2916	**−25.45**	**12.25**	**0.0390**	4.09	9.77	0.6761
Automobiles in the home	12.19	10.93	0.2653	−8.18	18.18	0.6531	**27.21**	**13.99**	**0.0528**

^a^Multi-level general linear model controlling for sex, and ethnicity with school as a random effect, unstandardized beta coefficients are presented. ^b^ISCOLE used a variety of measures to assess adiposity, all of which were significant in univariate analyses. To build the final models, stepwise addition was used to determine which measure of adiposity provided the closest fit for the data. Akaike information criterion, Bayesian information criterion, and level of significance were used to determine model fit. Waist circumference alone provided the best fit.

PA: physical activity; SE: Standard Error; TV: television.

NOTE: bolded data indicates significance (p<0.10).

**Table 4:** **Univariate correlates of self-reported screen time** ^**a**^

	**Total**			**Boys**			**Girls**		
**Variables**	**β-coefficient**	**SE**	***p-*** **value**	**β-coefficient**	**SE**	***p-*** **value**	**β-coefficient**	**SE**	***p-*** **value**
***Anthropometric*** ^b^									
Percent body fat	**0.01**	**0.00**	**0.0118**	**0.01**	**0.01**	**0.0677**	**0.01**	**0.01**	**0.0329**
Waist circumference	**0.01**	**0.00**	**0.0010**	**0.01**	**0.00**	**0.0325**	**0.01**	**0.01**	**0.0045**
BMI z-score	**0.07**	**0.03**	**0.0085**	**0.07**	**0.04**	**0.0930**	**0.09**	**0.04**	**0.0167**
***Behavioural characteristics***									
Unhealthy eating score	**0.13**	**0.03**	**<0.0001**	**0.08**	**0.03**	**0.0248**	**0.26**	**0.05**	**<0.0001**
Healthy eating score	**−0.11**	**0.03**	**<0.0001**	**0.07**	**0.03**	**0.0248**	**0.26**	**0.05**	**<0.0001**
Weekday breakfast consumption	0.07	0.18	0.7118	−0.21	0.41	0.6117	−0.12	0.21	0.5883
Weekend breakfast consumption	**−0.45**	**0.20**	**0.0250**	−0.09	0.27	0.7545	**−0.66**	**0.30**	**0.0257**
Commute to school	−0.09	0.06	0.1634	−0.02	0.09	0.8381	−0.14	0.09	−0.1384
Sleep quality	0.09	0.10	0.3687	**0.31**	**0.14**	**0.0309**	−0.05	0.13	0.6803
Sleep quantity	0.15	0.09	0.1055	**0.25**	**0.13**	**0.0507**	0.08	0.14	0.5728
Self-report PA	**0.13**	**0.06**	**0.0213**	**0.17**	**0.08**	**0.0427**	0.11	0.08	0.1880
***Family situation***									
Mother weight status	**0.19**	**0.06**	**0.0011**	0.10	0.08	0.2612	**0.29**	**0.08**	**0.0005**
Father weight status	0.09	0.06	0.1001	0.11	0.08	0.1678	0.10	0.08	0.2157
Mother education	**−0.15**	**0.08**	**0.0596**	−0.24	0.08	0.2731	**−0.19**	**0.11**	**0.0837**
Father education	**−0.35**	**0.07**	**<0.0001**	**−0.21**	**0.11**	**0.0459**	**−0.47**	**0.10**	**<0.0001**
Household income	**−0.19**	**0.06**	**0.0012**	**−0.24**	**0.08**	**0.0038**	**−0.20**	**0.08**	**0.0144**
***Home environment***									
# of TV’s in home	**0.15**	**0.06**	**0.0153**	**0.24**	**0.09**	**0.0090**	0.11	0.09	0.2041
TV in bedroom	**−0.32**	**0.07**	**<0.0001**	**−0.28**	**0.11**	**0.0093**	**−0.39**	**0.10**	**0.0002**
Automobiles in home	−0.00	0.12	0.9684	0.07	0.17	0.6630	−0.08	0.15	0.5696

^a^Multi-level general linear model controlling for sex, and ethnicity with school as a random effect, unstandardized beta coefficients are presented. ^b^ISCOLE used a variety of measures to assess adiposity, all of which were significant in univariate analyses. To build the final models, stepwise addition was used to determine which measure of adiposity provided the closest fit for the data. Akaike information criterion, Bayesian information criterion, and level of significance were used to determine model fit. Waist circumference alone provided the best fit.

PA: physical activity; SE: Standard Error; TV: television.

NOTE: bolded data indicates significance (p<0.10).

### Multivariate analyses

#### Total sedentary time

Results of the multivariate regression models for correlates of total SED are presented in Table 5. In the total sample, and for boys alone, SED was positively associated with waist circumference, and number of TVs in the home; SED was negatively associated with sleep quantity. For girls, SED was positively associated with waist circumference, and household automobile ownership.

**Table 5:** **Socio-ecological domains and final model for correlates of accelerometer measured SED and self-reported ST**

	**Total**			**Boys**			**Girls**		
**Variables**	**β-coefficient**	**Standard error**	***p-*** **value**	**β-coefficient**	**Standard error**	***p-*** **value**	**β-coefficient**	**Standard error**	***p-*** **value**
**Final model for total SED** ^a^									
Waist circumference	**1.54**	**0.31**	**<0.0001**	**1.38**	**0.48**	**0.0047**	**1.60**	**0.43**	**0.0002**
# TVs in home	**14.50**	**6.16**	**0.0190**	**25.33**	**9.75**	**0.0101**	-	-	-
Automobiles in home	-	-	-	-	-	-	**33.64**	**14.66**	**0.0225**
Unhealthy diet score	−2.49	2.69	0.3551	−1.75	4.39	0.6899	−1.02	3.56	0.7752
Sleep quantity	**22.29**	**9.16**	**0.0156**	**30.48**	**13.98**	**0.0305**	18.47	12.65	0.1455
**Final model for ST** ^**a**^									
Waist circumference	**0.01**	**0.00**	**0.0182**	**-**	**-**	**-**	**0.01**	**0.01**	**0.0257**
Mother weight status	**−0.10**	**0.08**	**0.0272**	**-**	**-**	**-**	**−0.17**	**0.08**	**0.0272**
Father education	**0.02**	**0.07**	**0.0062**	**-**	**-**	**-**	**0.32**	**0.09**	**0.0009**
# TVs in home	**0.13**	**0.06**	**0.0461**	**0.20**	**0.09**	**0.0260**	**-**	**-**	**-**
TV in bedroom	−0.14	0.08	0.0793	**−0.29**	**0.12**	**0.0146**	−0.09	0.10	0.3680
Unhealthy eating score	**0.13**	**0.03**	**<0.0001**	**0.07**	**0.03**	**0.0310**	**0.21**	**0.05**	**<0.0001**
Healthy eating score	**−0.10**	**0.03**	**0.0001**	**-**	**-**	**-**	**−0.10**	**0.04**	**0.0059**
Weekend breakfast consumption	**−0.77**	**0.20**	**<0.0001**	**-**	**-**	**-**	**−0.93**	**0.30**	**0.0021**

^a^Multilevel general linear model including all significant variables from the full model, controlling for sex, ethnicity, household income, and number of siblings with school as a random effect.

PA: physical activity; TV: television.

NOTE: bolded data indicates significance (p<0.05).

#### Screen time

Results of multivariate regression models for correlates of self-reported ST are presented in Table 5. In the whole sample, ST was positively associated with waist circumference, mother’s weight status (classified overweight or obese), father’s education (greater than high school), number of TVs in the home, and unhealthy eating pattern score. ST was negatively associated with healthy eating pattern score and weekend breakfast consumption. In boys, ST was positively associated with number of TVs in the house, presence of a TV in the child’s bedroom, and unhealthy eating pattern score score. For girls, ST was positively associated with waist circumference, mother’s weight status (classified as overweight or obese), father’s education (higher than high school), and unhealthy eating pattern score; negative associations were found with healthy eating pattern score and weekend breakfast consumption

## Discussion

This study aimed to identify correlates of accelerometer measured SED and self-reported ST in 10 year-old Canadian children. In the total sample, common correlates of SED and ST included waist circumference (positive association) and number of TVs in the home (positive association). In the total sample, increased SED was also associated with poor sleep quantity. We identified a greater number of correlates of ST than for SED. Specifically, correlates of ST in the total sample also included mother’s weight status (negative association), father’s education (positive association), unhealthy eating pattern score (positive association), healthy eating pattern score (negative association), and weekend breakfast consumption (negative association). Correlates identified here are similar to those identified in previous studies [11]. However, this is one of few studies to identify correlates of both SED and ST in the same population, and the first to examine this in a sample of Canadian children. This information can be used to help inform public health strategies and messages. Specifically, results from this work suggest that to reduce both SED and ST, public health messages (and interventions) focus on healthy weights, and reducing the number of TVs present in the house.

A positive association between waist circumference, and both SED and ST is consistent with previous work in boys and girls [27], and in both younger [27] and older [28] children. Although the cross-sectional nature of ISCOLE cannot provide information on causality, findings from a previous study show that fat mass is a significant predictor of sedentary time, but sedentary time is not a predictor of fat mass in children of the same age [29]. This is consistent with numerous other studies that have shown a significant relationship between higher weight status/adiposity and higher levels of sedentarism in children and in adults [4,30]. Recent work in adults has shown that individuals with high accelerometer measured SED, and individuals with high self-reported ST are more likely to have metabolic syndrome compared to those in the lower SED and ST groups [31]. We also saw that mother’s weight status (being overweight or obese) was a predictor for higher ST in girls, but not boys (p < 0.0001, data not shown).

Our work showed a significant relationship between unhealthy/healthy eating pattern scores and ST, but not with SED. This may be partly due to increased energy intake, primarily through energy dense snacking, while watching TV [32]. Previous work has shown that both boys and girls who report high levels of reading or homework consume significantly less percent of energy from fat that those who reported low levels of reading or homework [33]. Consistent with previous work [33,34], we showed that higher scores unhealthy eating pattern (i.e., a *more* unhealthy diet) and lower scores for healthy eating pattern (i.e., a *more* healthy diet) are correlates of ST for boys and girls. Previous research from Utter et al. showed that girls and boys who reported the highest screen time consumed 300–400 calories more per day than those who reported the lowest screen time [33]. Although we are unable to comment on the quantity (i.e., number of calories) of energy intake, the association of ST with consumption of energy-dense foods, such as soft drinks, fried food, and unhealthy snacks, is consistent with current results, as well as other work using the ISCOLE Canada dataset [35].

For SED, potential correlates (except weight status and healthy/unhealthy diet scores) were re-coded as dichotomous variables. The advantage of this approach was that all variables were on approximately the same scale, so that the strength of the association of each correlate could be examined, and interpreted as a proportional increase in the outcome variable (e.g., a unit increase in the beta coefficient for a correlate of SED, represented an approximate increase of one minute per day of SED) (Table 5). This information can help inform public health interventions to reduce SED. Poor self-reported sleep quantity represented an approximate increase of 22.3 minutes of SED per day, and having more TVs in the home represented an approximate increase in 14.5 minutes of SED per day. Because ST was measured as a score, instead of hours per day, it could not be interpreted the same way, but strength of association can still examined. Not eating breakfast on the weekends and having two or more TVs in the house was associated with the largest increase in ST score.

Self-reported number of days being physically active for greater than 60 minutes was not a correlate of SED or ST. To understand the significance of this finding, we re-ran the models to include accelerometer-derived variables (intensities as well as average counts per minute) instead of the self-report physical activity (data not shown). When included, LPA, MVPA, and total accelerometer activity counts were all found to be significant correlates of SED, but not for ST. This was true when including the movement variables by themselves, or when including combinations of different intensity levels. There is debate as to whether to include more than one movement variable in the same model because although they may not be highly correlated (i.e., no issues with multicollinearity), they are also dependent on each other since they are derived from the same measurement device (i.e. proportional error). Including accelerometer-derived activity variables can also mask variance that could be attributed to other potential correlates.

Consistent with previous work, we found that self-reported ST explained only a small portion (approximately 33%) of accelerometer measured SED [10]. ST did have an upper limit of 10 hours per day (5 hours of TV viewing and 5 hours of video/computer games) but few participants reported >5 hours of TV per day (3% and 9% on weekdays and weekend days, respectively) or >5 hours of computer/video games per day (1% and 6% on weekdays and weekend days, respectively). While accelerometers are able to provide accurate information on activity for the whole day, they are not able to detect the difference between some activities (e.g., standing still versus sitting still), and are not able to provide any context for the behaviour (e.g., reading versus watching TV) [36]. This suggests that there is a large proportion of daily SED that is unaccounted for by measuring ST alone. Future work should aim to understand correlates, and related health effects (both positive *and* negative) of other, non-screen based sedentary behaviours (e.g., reading a book, passive transportation).

Future work should also aim to understand how technology influences the use of electronic devices in daily life. This is important for health care providers when planning public health strategies to reduce SED and ST, and for sedentary behaviour researchers when defining SED and ST. For example, with the advent of smart phones, and tablet computers, sedentary multi-tasking is common; a child is able to watch TV, play on the computer, and use a smart phone at the same time. Further, a device can be multi-purpose (e.g., can be used as TV, video game, book, musical instrument, computer, etc.). We know very little about how this affects healthy growth and development. Future work needs to better understand possible associations (negative or positive) with both screen- and non-screen based behaviours to help inform public health messages.

This paper has both strengths and limitations. Accelerometers have been shown to be a valid tool to measure activity of all levels of intensity. However, it is well-understood that a hip-placed monitor is less effective in distinguishing sedentary postures, like lying or sitting, from other very light intensity activities performed while standing; further, accelerometers cannot accurately capture upper body movements, cycling, or activities when the monitor is removed. For ISCOLE, algorithms were used to distinguish sleep from SED. The algorithms have been validated and published [37], and accelerometer cut points for SED have high sensitivity [19], but there is always the possibility of misclassification. Many potential correlates of SED and ST were based on child- or parent-report, which is subject to either over- or under-reporting. It is also possible that these correlates are unique to Canadian children in the current study, and caution should be used when generalizing these results to other populations. Further, it would have been beneficial to include additional potential correlates related to the family environment (e.g., parenting rules and restrictions relating to ST, parental ST habits) [38]. Finally, causality of correlates identified here cannot be determined from cross-sectional data and future studies using a longitudinal design will be needed to address this issue.

## Conclusion

This study aimed to identify correlates of objectively measured total SED and self-reported ST in Canadian children. For both SED and ST, some of the correlates we identified are easily modifiable (e.g., reducing the number of TVs present in the home), while others may require more intense behavioural interventions (e.g., reducing waist circumference). Overall, we were able to identify a wider variety of correlates of ST than of SED. Future work should examine the relationship between a range of sedentary behaviours, including non-screen based pursuits, across a more geographically and culturally distinct population to inform public health strategies and messages.

## Abbreviations

BMI: Body mass index
LPA: Light-intensity physical activity
MVPA: Moderate- to vigorous-intensity physical activity
PA: Physical activity
SED: Accelerometer measured total sedentary time
ST: Self-report screen time
TV: Television
